# “I know why I am taking this pill”: Young women navigation of disclosure and support for PrEP uptake and adherence in Eastern Cape Province, South Africa

**DOI:** 10.1371/journal.pgph.0000636

**Published:** 2023-01-20

**Authors:** Joseph Daniels, Lindsey De Vos, Dana Bezuidenhout, Millicent Atujuna, Connie Celum, Sybil Hosek, Linda-Gail Bekker, Andrew Medina-Marino

**Affiliations:** 1 Edson College of Nursing and Health Innovation, Arizona State University, Phoenix, Arizona, United States of America; 2 Research Unit, Foundation for Professional Development, Buffalo City Metro, Eastern Cape Province, South Africa; 3 Department of Epidemiology, Mailman School of Public Health, Columbia University, New York, United States of America; 4 The Desmond Tutu HIV Centre, University of Cape Town, Cape Town, South Africa; 5 Departments of Global Health, Medicine and Epidemiology, University of Washington, Seattle, WA, United States of America; 6 Departments of Psychiatry and Infectious Disease, Stroger Hospital of Cook County, Chicago, IL, United States of America; 7 Department of Psychiatry, Perelman School of Medicine, University of Pennsylvania, Philadelphia, PA, United States of America; American University, UNITED STATES

## Abstract

There is limited understanding of the dynamic interplay between adolescent girl’s and young women’s (AGYW) disclosure and social support for using oral pre-exposure prophylaxis (PrEP) and adherence. Towards this, we conducted interviews with 42 AGYW enrolled in The Community PrEP Study who exhibited either high or low blood concentrations of tenofovir-diphosphate (TFV-DP) in dried blood spots. Guided by Theories of Practice, interviews and analysis focused on AGYW perspectives and experiences with PrEP disclosure, support and adherence. AGYW with high TFV-DP blood concentrations described larger social support networks and disclosure events. In contrast, those with low TFV-DP blood concentrations described disclosing to fewer people, resulting in limited social support. Participants discussed partner support, however, this support was not described as consequential to adherence, irrespective of TFV-DP levels. Those with high levels of TFV-DP in their blood described the ability to navigate social scrutiny and changes in social support, while those with low levels of TFV-DP in their blood were more likely to question their own continued use of PrEP. To facilitate AGYW’s prevention-effective use of PrEP, expanded skill-building for disclosure and resiliency against changes to social support should be examined as part of PrEP services.

## Introduction

Globally, adolescent girls and young women (AGYW) bear a disproportionate burden of new HIV infections [1,2]. HIV pre-exposure prophylaxis (PrEP) is highly effective for preventing HIV infection among AGYW with high, but not perfect, adherence [3]. While access to PrEP continues to increase [4–6], its prevention-effective use remains low [7], resulting in sub-optimal benefits and protection among those using PrEP [3]. Consequently, improving our understanding of the factors that influence AGYW’s prevention-effective use of and adherence to PrEP is integral to decrease HIV incidence, especially among AGYW.

In South Africa, where HIV incidence and prevalence among AGYW ages 15–24 is unacceptably high [8], HIV prevention decision-making is strongly influenced by transactional sexual relationships [9], having concurrent or older sexual partners [9,10], limited agency in relationships [11,12], and interpersonal violence [13,14]. Given these significant risk factors, biomedical prevention options that are under an individual’s direct control, such as PrEP, and not subject to partner negotiation, are essential to reducing HIV incidence among AGYW [15–17]. Unfortunately, AGYW taking PrEP are more likely to experience unintended disclosure of sexual activity, and encounter related conflict that leads to the loss of support, as well as physical and/or mental harm [18,19]. Shaming of AGYW by partners and family, many who may not fully understand her HIV risk, can cause young women to discontinue their PrEP use [20–22]. When young women do remain on PrEP, anticipated negative reactions, including mistrust, stigma and relationship dissolution, often results in PrEP secrecy and low adherence [19,23,24]. Alternatively, disclosure to family members, partners and other trusted individuals can minimize negative reactions and stigmatization, and improve PrEP uptake and adherence among AGYW. However, support after disclosure is not static or unchanging once garnered [8,9], and there is limited understanding of how young women continually navigate social networks in order to generate and sustain support to achieve prevention-effective use of PrEP.

The Community PrEP Study (CPS), a mixed methods study conducted in South Africa’s Eastern Cape Province, leveraged community-based platforms to increase access, uptake and adherence to PrEP for AGWY [25–28]. Nested within CPS, we qualitatively compared disclosure and support experiences among AGYW with high and low tenofovir-diphosphate (TFV-DP) concentrations in dried blood spots (DBS) [25]. Ultimately, we sought to elucidate the dynamic relationships between a young woman’s disclosure of PrEP use, social support and adherence to PrEP, and how these change overtime, with an eye towards improving interventions to support AGYW’s prevention-effective use of daily oral and, potentially, long-acting PrEP.

## Methods

This qualitative sub-study of once-off in-depth interviews with AGYW was nested within the larger Community PrEP Study; a full study protocol of CPS has been previously published [25]. Briefly, CPS sought to assess the acceptability and feasibility of leveraging community-based HIV counselling and testing services (i.e., mobile tent testing and door-to-door home-based testing) to identify and offer at-risk AGYW same-day access to and initiation on oral PrEP; given the likelihood of social desirability bias, structural risk factors, and the dynamic nature of HIV risk among South African AGYW, prior sexual activity was not used as inclusion/exclusion criterion [25,29–31]. AGYW were informed about PrEP following a negative HIV test, and referred to community-based PrEP initiation sites. Following PrEP initiation, AGYW were randomized to one of three community-based behavioral interventions (intervention arms: health club vs. one-on-one adherence support; control: community-based medication refill) aimed at improving adherence and optimizing prevention effective use of PrEP. This qualitative sub-study investigated the dynamic relationships between a young woman’s disclosure of PrEP use, social support and adherence to PrEP, and how these change overtime; previous work has described the importance of PrEP to study participant’s and their reasons for starting PrEP [26].

### Study location

CPS was conducted in the Ndevana (rural) and Scenery Park (peri-urban) communities in Buffalo City Metro (BCM) Health District, Eastern Cape, South Africa. BCM has an estimated population of 834,977 persons (Black Africa = 85.1%), an estimated total population HIV prevalence of 12.4% and incidence of 0.54% per year, and an estimated youth (age 15–24 years) HIV prevalence of 8.5% and incidence of 1.66% (female youth = 12.8% prevalence, 2.4% incidence; male youth = 4.2% prevalence, 0.94% incidence) [32].

### Participant selection & inclusion

AGYW actively enrolled in CPS and with a valid measure of intracellular tenofovir-diphosphate (TFV-DP) concentration in their blood extracted from red blood cells in dried blood spots (DBS) at 6-months post-PrEP initiation were eligible for inclusion in this nested sub-study. Purposive sampling of participants was conducted to ensure equal representation of the following characteristics: 1) urban vs. rural study site, 2) one-on-one vs. health club study arms, and 3) high vs. low levels of TFV-DP in DBS. DBS were processed and analyzed by the Clinical PK Pharmacology Laboratory, University of Cape Town [33]; per the iPrEX OLE STUDY, High adherence was defined as ≥700 fmol/DBS punch (~4–7 tablets per week) and low adherence was defined as ≤699 fmol/DBS punch (~3 or less tablets per week). All interviews were conducted within 4–8 months of DBS [34].

Participants were contacted outside of normal study visits and invited to the study site to learn more about the qualitative interview. Upon presentation, participants were provided a summary of the qualitative interview topical areas, asked to provide written informed consent, if interested in participating, and immediately interviewed by research staff. Participants received a snack and R100 (~$7 USD) for their time and travel.

### Theory of practice (ToP) for PrEP

We use ToP to examine and frame intersecting factors in AGYW PrEP engagement; a framework previously used to understand AGYW’s engagement and disengagement from HIV services in sub-Saharan African settings [35,36]. In ToP, practices are influenced by their individual and social meaning, available resources, individual competency and assertion of agency, and other practices within constraining social norms and intersecting risks [12]. In the context of PrEP, ToP can be used to examine and frame intersecting factors that influence PrEP uptake, initiation, prevention effective use and disengagement [15,35,36]. Specifically, not only does PrEP knowledge and self-management skills influence AGYW adherence, but also their motivation and agency to do so hinges on other factors like perceived HIV risk, community PrEP knowledge and norms of use, and other daily activities [35]. Given that PrEP is a relatively new HIV prevention tool in Eastern Cape Province, social norms and values relating to PrEP use by AGYW in this setting are still developing. This may, in turn, create barriers or motivators for prevention of effective use of PrEP [12,37]. In addition, other practices may influence PrEP use, including school schedules, staying with boyfriends and friends, and familial living arrangements and expectations [35]. The ability for AGYW to manage their PrEP in these contexts directly influences adherence. However, the degree to which AGYW can integrate PrEP into their lives, navigate environments with differing degrees of support, and exhibit resiliency in their PrEP use is not fully understood.

### Data collection

Between March and July 2020, participants completed in-depth interviews led by trained female study staff members that were highly familiar with the study communities and local socio-cultural dynamics. Senior members of the qualitative research team conducted a 3-day interactive training with interview staff on qualitative research, interview methods and the interview protocol, including observation, field notes, probing and prompting techniques. Interviewers all had experience with previous health research studies, qualitative interviews, and human research ethics. Interview guides examined four general domains: 1) daily routines of PrEP storage and usage, 2) influential community settings (i.e. school, church, households), 3) disclosure experiences and decisions over time, and 4) support development and changes over time with a focus on family members, friends, and partners. All interviews were conducted in the participants preferred language (i.e., isiXhosa or English) and were complemented with observation guides. Interviews were approximately 60 minutes in length, and were audio-recorded, directly transcribed, and then translated into English. For quality control purposes and prior to analysis, transcripts were reviewed by members of the study data team. The core research team (Daniels, Medina-Marino, De Vos, and Bezuidenhout) held weekly study meetings, to monitor and refine data collection processes.

### Data analysis

Interviews were transcribed directly into the language in which the interview was performed; English interviews were directly transcribed into English, isiXhosa interviews were transcribed into isiXhosa and then translated into English. Data were analyzed using a deductive approach guided by theories of practice in that at each step of analysis we examined participant’s perspectives on their behaviors and social networks their influence on PrEP use [35]. The main analytical focus was on the influence of family, partners, and friends on PrEP use, and then the degree to which AGYW exerted their agency relating to PrEP adherence [15,36]. We further examined disclosure experiences and how changes in support influenced PrEP use over time. PrEP disclosure events were operationalized in transcripts when participants discussed their PrEP use with: 1) family members, 2) partners, or 3) friends. Context of disclosure events included details around how, where and when a participant disclosed their PrEP use, disclosure outcomes, and the impact of disclosure on the participant PrEP use and their social network. Disclosure events were compared by PrEP adherence level (low and high DBS).

Coding and preliminary data analysis occurred throughout data-collection. A sub-set of translated transcripts were open coded by three members of the research team. Codes were then defined, refined, and organized mutually into a codebook through regular meetings. To identify emerging themes, preliminary analysis was performed on the first half of completed transcripts. The final codebook was then applied to all transcripts. After coding was completed, memo writing and matrices were used to examine interactions and interdependencies between PrEP disclosure, support, adherence levels (high vs. low adherence), study sites (urban vs. rural) and settings (households, schools, and with boyfriends and friends), and partnership status. Additional matrices examined common and divergent disclosure and support changes over time. Findings were presented to the larger research team (all authors) for feedback and additional guidance on analysis.

### Ethics approval

Ethical considerations and trial registration PrEP has been approved for use by international and domestic governing bodies in South Africa. Written informed consent was obtained from participants at multiple time points, including prior to baseline questionnaire, PrEP initiation, and all qualitative interviews. The study protocol has received full ethical approval from the University of Cape Town Human Research Ethics Committee (Ref: 289/2018) and has been registered with ClinicalTrials.gov (NCT03977181). Participation by AGYW under 18 years of age without guardian consent received Ministerial Consent for Non-Therapeutic Research with Minors from the Eastern Cape Department of Health, which is part of the approval above (Ref: 289/2018).

### Participant representation

In-text quotes are representative of all participants by adherence category; S1 Table displays basic information of the AGYW for whom we present in this manuscript (S1 Table). These data include PrEP adherence behavior based on DBS, age, and recruitment site. Also, in this table, the names listed are not the actual participant’s names, but a study identifier to humanize our participants when presenting quotes.

## Results

### Description of study participants

A total of 42 cis-gender AGYW, aged 16–24 years, were interviewed. Sixty-eight percent reported ever having a sexual partner, 58% reported having a current primary sex partner, and half reported having sex within the last four months. Most participants (85%) were living at home with their parents or grandparents. Those categorized as having high levels of adherence had a median TFV-DP concentration of 839.5 fmol/DBS punch (range = 776–2880 fmol/DBS punch), while those categorized as having low levels of adherence had a median TFV-DP concentration of 93.8 fmol/DBS punch (range = 0–489 fmol/DBS punch).

### Choosing to disclose to one or many

A majority of participants discussed disclosing their PrEP use to another female family member first, most often their mother, and other females consequential to their adherence behavior. However, this differed by the degree to which they anticipated a negative reaction which could derail their subsequent PrEP use.

Young women with low blood levels of TFV-DP often discussed apprehension about disclosure, anticipating negative reactions like being told to stop taking PrEP:

*I was scared thinking that she [mother] might force me to stop like other parents did to their children… At first*, *I kept taking it in secret because I still did not know if she [mother] wanted me to continue or stop… It affected me badly because I was mostly quiet about PrEP even when my friends were telling others about PrEP*, *telling them [other women] to come [to the study]*… *[Asemahle*, *18 years of age]*

Parents were perceived as disapproving PrEP use, and it was expected that they would tell their daughters to quit. Keeping their PrEP use secret was a strategy for AGYW, leading to increased sense of isolation from family and friends. This anticipated PrEP disapproval led some young women to expect intense questioning after disclosure: *“cause I thought she will react funny like*, *‘What is this pill*? *How do you trust these people*?*’ But*, *she reacted right [allowed her to continue]*, *and I continued with it [PrEP] then” (*Babalwa, 16 years of age). Although most young women with low blood levels of TFV-DP did describe disclosing their PrEP use, it was to only one or two people, mostly those that they lived with like parents or grandmothers: “*My mom knows about PrEP*, *but my dad doesn’t [And] It’s not free to say everything in front of my brothers*” (Ndiliswa, 22 years of age). This singular support was often described as permission to continue taking PrEP with few references of motivation and encouragement received from parents or other family members.

Conversely, AGYW with high blood levels of TFV-DP were more likely to discuss their PrEP use with key people in their lives, and the type of support received from these key individuals. When asked about whom they discussed their PrEP use, some young women responded:

*Firstly*, *I talked with my cousins and my family members*. *And*, *then in Q [town in Eastern Cape]*, *I do have friends [that I discuss it with] and then mostly the sisters of my boyfriend… because I want them to be able to protect their [them]selves*. *[Avela*, *22 years of age]*

*People who are close to me support me very much in things that are related to PrEP*. *They remind me [to take my PrEP] and ask if I’m still continuing [with PrEP]*, *and I say*, *‘Yes*.*’ And they ask*, *‘How is it treating me*?*’*, *and I say*, *‘There’s nothing [no side effects]*.*’ I’m happy because most of them are people who are important to me*. *So*, *when supported by people who are important to you*, *just say*, *‘Let me go [pick up my PrEP] because they are important*, *and they support me*.*’ [Zintle*, *19 years of age]*

Many young women with high TFV-DP concentrations spoke about how PrEP will serve their future by protecting them, as well as protecting other young women close in their social network by educating them about PrEP and how it prevents HIV infection. Among high adherers, disclosure narratives were often grounded in existing relationships, which involved close family members not only reminding them to take PrEP but also asking about her experience, which motivated their adherence. Some young women described people who supported them in other areas of their lives, as discussed:

*“My mother*, *[I] talked to her the first day I heard about PrEP…There is a teacher who loves me and stands by the girls*. *[The school] called us [to a meeting]*, *so all three of us [sisters/siblings] [could] learn about PrEP…*. *I told my friends about PrEP*, *and I did take it too*.*” [Lulama*, *17 years of age]*

Many participants advocated for PrEP to other girls and women as a strategy to generate support within their social networks when they wanted to initiate PrEP.

### Boyfriend support is additive but not consequential

Support from boyfriends was frequently discussed among all young women in our study irrespective of their adherence level. A common narrative emerged that boyfriends had multiple partners, as did some of the young women. However, their support was discussed as an added benefit only, but not essential to their decision to take PrEP or their adherence.

Among those with high TFV-DP concentrations, boyfriends were often described as supportive by motivating uptake and dosing as well as expressing interest in PrEP for themselves. When disclosing their PrEP use to their boyfriends, there was a basic discussion about why they were taking PrEP, followed by the boyfriend agreeing or supporting the decision:

*He asked*, *‘Why I used it [PrEP]*?*’ And*, *I told him that*, *‘You guys*, *[we girls] cannot trust you fully*. *You are not faithful…*. *That is why I’m using it*.*’ He said*, *‘It’s the right thing [taking PrEP]’*. *He was interested [in PrEP]*. *He said*, *‘Why don’t you give me one*?*’ I said*, *‘No*, *these pills are for the whole month [for me]*, *and so I can’t give it to you*.*’ [Lindelwa*, *20 years of age]*

A common perspective among young women in this study was that men can’t be trusted in general, and some boyfriends agreed with this assessment and supported their girlfriend’s decision to take PrEP. Further, some men were interested in taking their girlfriend’s PrEP after learning about the benefits of PrEP, but there is limited evidence such sharing actually occurred. Rather, young women explained that the PrEP was them only. Other boyfriends provided support through dosing reminders and monthly medication collection reminders: *“He [boyfriend] was asking if I have gone to the site [for medication collection] and is always reminding me about my date and asks when I’m going” (*Zintle, 19 years of age). Multi-dimensional support from boyfriends was reported by many participants with high blood levels of TFV-DP.

*When I come [back from my sessions]*, *he asks what I did there and what do they [study staff] ask*? *What is the reason behind PrEP*? *There was the time where my pills were finished*, *and I came here [study site] and it was already closed*. *So*, *I didn’t have pills for the weekend*. *So*, *he [boyfriend] asked if I could get it [PrEP] at the chemist so that he can buy it for me*, *and I said yes*, *and I told [him] how much it cost*. *[Anathi*, *24 years of age]*

Among young women who had a supportive boyfriend, they often received encouragement to attend sessions and offering to pay for PrEP outside the study. However, young women also said that they couldn’t rely on their boyfriends for consistent support: ‘*if ever I put my health on other person’s hands*, *I will be wrong*’ *[Anathi*, *24 years of age]*.

Although boyfriends were often described as less supportive by women and girls with low blood levels of TFV-DP, this did not dissuade them:

***Noziphiwo (18 years of age)***: *I told him [boyfriend about me taking PrEP]*, *but he did not want me to use it [PrEP]*. *He was asking*, *‘What pill is this*?*’ He was [acting] the same as those people who spoke negative things about PrEP*, *saying ‘No*, *you are infecting yourself with the [HIV] virus*!*’ What pill is protecting you from something you don’t have*?*’*

***Interviewer***: *The way that your boyfriend reacted*, *how did it make you feel*?

***Noziphiwo***: *I didn’t care about him because I know why I am taking this pill*.

Some partners were suspicious and tried using misinformation and conspiracy theories to try to get them to stop taking PrEP. Despite this, most young women persisted in taking PrEP to protect themselves against infection.

Regardless if participants had high or low blood levels of TFV-DP, some anticipated negative reactions from partners that may have threatened their PrEP use. As a result, some participants decided not to disclose their PrEP use, and thus exclude their boyfriends from their support network.

*I was afraid [to tell my boyfriend about PrEP]*. *I thought he will say [that] I must stop taking it*. *[Akhona*, *17 years of age]*.

*I did make a plan [to disclose to my boyfriend] but I wanted to see first how he [boyfriend] thinks about PrEP*. *If I’m talking about important things*, *I want to see how they react or think*. *When I saw that this person [boyfriend] is not thinking like I do*, *there is no way that he will understand what I will tell him [about PrEP]*. *[Cwayita*, *23 years of age]*

Often women assessed their partner’s openness to PrEP before disclosing. If they anticipated a negative reaction or their boyfriend/partner was not open to concerns important to her, these young women would not disclose to them.

### Navigating scrutiny and support loss

Several participants described a heightened sense of scrutiny from family and community members while taking PrEP. This influenced adherence for some but not all young women, as using PrEP signaled to others that the participant was sexually active or had a partner. Many young women with low blood levels of TFV-DP described this perceived or enacted scrutiny as significantly influential:

*If I want do something*, *there is always someone watching*, *and they’ll be like*, *‘She’s doing that [PrEP and having sex]*? *Why is she doing this*? *Why is that*, *why is that*? *It’s mind draining because when we’re doing something [PrEP] we have to be like*, *‘Ok*, *someone is watching*. *I must not do that [PrEP and having sex]*? *I must let myself do this*?*’ Because everyone sees you as this person*, *so it’s mind draining*. [Siyanda, 18 years of age]

Perceived family and community negative impressions of young women taking PrEP impacted adherence. Young women reported being exhausted and unnerved by negative perceptions of them taking PrEP, leading them to think that they may have made the wrong decision. Further, most young women were uncomfortable that others assumed that they were having sex. Such draining scrutiny deterred some young women from continuing with PrEP after initiating:

*You take PrEP when you know that you have a partner*, *and they [community members] will be like*, *‘Now*, *you are having sexual intercourse*.*’ It won’t be easy for them [young women] to come [to the study site] because*, *even when you go to the clinic for HIV prevention*, *people look at you funny and be like*, *‘You’re also do[ing] these things*?*’ So*, *that’s why some young women would never come to collect PrEP [at the study site]*. [Siyanda]

Scrutiny also included criticism from others like family:

*At home*, *they were discouraging it [PrEP] because they said*, *‘How can there be a prevention for something that has no cure*? *It’s not possible*.*’*. *I didn’t mind because I was expecting criticism*…*but they were laughing at me saying*, *‘I will get sick from taking something that I don’t even know*.*’ [Nomble*. *17 years of age]*

Family criticism was highly influential among those with low blood levels of TFV-DP. This criticism was largely based on not understanding how PrEP worked, and in some cases, mistrust of HIV-related medicine. This familial push-back and criticism for taking PrEP, especially from those whom young women were seeking support, was a common experience among participants with low blood levels of TFV-DP after they disclosed. Those with high blood levels of TFV-DP also encountered familiar push-back and criticism. However, they were able to navigate this push-back and key supporter loss, and continued taking PrEP.

***Interviewer***: *Is there any bad experience [that you’ve had] when taking or using your PrEP?*

***Lindelwa*** *(20 years of age)*: *I think it’s bad because before she (aunt) talked with her friend*, *she was supportive*. *Like with my dates*, *I told her my PrEP dates*, *and asked her to remind me*, *if I forgot*. *She phoned me even if I’m at school*. *And even if I’m home*, *she asked*: *‘Did you take your pills*?*’ But*, *after she talked with that person*, *she stopped supporting me…She didn’t support the thing that is about PrEP*, *because she heard the pill makes you sick [Gives you HIV]*, *if you take it while you are not sick*.

***Interviewer*:** *Does anyone know about your action plan [PrEP dosing plan]?*

***Lindelwa*:** …*My roommate last year, she knew. If I went out on a rush, she’d asked, ‘Hey, did you take your pills?’ I told her that I hope this ringing alarm was not disturbing her because it’s for my pills [pill dosing reminder system]*.

**********

**Fundiswa** (16 years of age): *I told him [about PrEP]*, *and he did not believe it*. *He thought it is ARVs because he heard that they [pills] are big*. *So*, *he thought I am ARVs*. *I dumped him*.

**Interviewer**: *So*, *your relationship ended because of PrEP*?

**Fundiswa**: *Yes*, *because he said I am taking ARVs*, *and I am hiding it*.

**Interviewer**: *How did it make you feel*?

**Fundiswa**: *I was very hurt*. *I even told my family about it*, *and they said I did right by dumping him*. *They knew about our relationship*, *and so did his family… [my family said that] ‘if he wanted to know about your status*, *he would have come here and asked about it*.*’*

Among those with high blood levels of TFV-DP, young women were able and desired to navigate loss from key and other supporters. Some young women experienced changes in supporter perspectives of PrEP, from being supportive to unsupportive due to misinformation within their social network. Although family members wanted them to stop, these young women persevered and continued their PrEP use by finding support among friends. Further, others decided to end relationships because supporters or partners didn’t believe that they were using PrEP as an HIV prevention tool, and not treatment, even after attempting to educate them. Most young women with high blood levels of TFV-DP discussed these changes in support and the ability keep the perspective to navigate these dynamics over time: *‘*…*today your mother supports you*. *Tomorrow your dad supports you*. *The following day your brother supports you and your mother has stopped being supportive*. *Like it changes…’ [Bongani*. *23 years of age]*

## Discussion

The prevention-effective use of PrEP, especially among AGYW, remains low due to ongoing social barriers, stigma, and social norms and poor social network support [1,2]. Realizing the promise of oral PrEP is not just about increasing its access and improving its uptake, but about identifying the intersecting, influential factors that impact its prevention-effective use among AGYW [36]. Through the lens of ToP, we could identify these influential factors in PrEP use and then describe how these are inter-related to influence AGYW’s decisions for PrEP engagement or disengagement in their lives [35].

We found that in order to generate support for their PrEP use, AGYW make choices about to whom they will disclose their PrEP use [1,11,12]. Nearly all participants in our study discussed disclosing their PrEP use to at least one of three cadres of individuals in their social support networks: family, friends, and partners. Such disclosure is often associated with the need to navigate HIV-related stigma, which may hinder their prevention effective use of PrEP [2,38–40]. We found salient differences between those with high and low levels of TFV-DP in dried blood spots with regards to disclosure and support narratives, how participants navigated scrutiny and loss of supporters, and how they deployed HIV knowledge sharing to build PrEP value within their social networks.

AGYW in our study described assessing potential supporter’s receptiveness to learn about and discuss PrEP for HIV prevention. Based on this, AGYW selectively disclosed their PrEP use to key individuals in their social networks in order to gain support for the effective integration of PrEP in their lives. Towards this, AGYW who disclosed their PrEP used to individuals across all three social network groups (i.e., family, friends, and boyfriend/partner) were found to have high blood levels of TFV-DP. Furthermore, those with high blood levels of TFV-DP discussed compartmentalizing the support they received based on their needs for motivation, dosing reminders, emotional connectedness and validation of their PrEP use. In comparison, those with low blood levels of TFV-DP disclosed to individuals in two or fewer of these social network groups, and were unable to compartmentalize the support they needed [41]. These AGYW had social networks that were too small, and/or were unable to effectively identify supporters, and/or experienced too many barriers to developing a shared value of PrEP use amongst their potential supporters. Such limitations may have increased their isolation with regards to taking PrEP, and thus reduced effective dosing behaviors. Ultimately, the ability of AGYW to assess their needs to consistently use daily oral PrEP, and then access support from different people, was a resilient strategy that not all young women were capable of managing [12,15].

The ability to compartmentalize support played a significant role in these AGYW’s responses to community perceptions of their PrEP use, and their resiliency to HIV-related stigma. Most participants described being scrutinized and criticized by those they sought support from. One participant described this scrutiny as “draining,” as they had to constantly defend their PrEP use against assumptions that they were already living with HIV. This made AGYW in our study and others further feel untrusted by their communities and social networks, and resulted in them questioning their decisions about having a sex life and taking PrEP [2,39,42,43]. Interestingly, we found AGYW describing distinct responses to this criticism and scrutiny. Specifically, those with low blood levels of TFV-DP were unable to identify alternative supporters when they experienced this criticism, which may have influenced their PrEP (dis-)engagement. In comparison, those with high TFV-DP blood levels described the ability to continuously identify, disclose, navigate and recoup multi-dimensional support (i.e. emotional support, medication reminders, shared value relating to PrEP use) from individuals representing all three cadres of supporters. These distinct responses seemed dependent on an AGYW’s ability to secure support for their PrEP use and their assessment of the future benefits of PrEP to their life and career goals, which was a motivator for AGYW with high blood levels of TFV-DP.

Strengths of our study included the large sample size, sampling of participants from both urban and rural communities, and the use of validated biomedical measures to assess adherence. However, the time difference between when the biomedical measure of adherence was conducted and when an interview was conducted is a limitation, as disclosure, support and adherence experiences described during one’s interview may have been different from when adherence was measured. Furthermore, we were only able to interview AGYW remaining in the study, thus the experiences of those that discontinued PrEP were not captured in our study. Finally, extrapolation of our finds to the larger South African context is limited given that our data were collected in one setting in South Africa.

## Conclusion

AGYW from Eastern Cape Province, South Africa, with high and low blood levels of TFV-DP helped to elucidate how access to multidimensional support from key individuals and social network groupings may be associated with prevention-effective adherence to PrEP. As oral and long-acting injectable PrEP formulations are rolled out in South Africa, and globally in other high HIV risk settings, adherence support interventions must consider how intersecting socio-ecological domains influencing PrEP use. Community-based awareness campaigns are needed to facilitate social norms for PrEP use. Furthermore, interventions should integrate skill-building to 1) navigate changes to support, and 2) how to compartmentalize and access the social and emotional support the need. Such tools may foster AGYW’s agency in their health decisions and behaviors, improve and sustain their prevention-effective use of PrEP, and thus reducing their risk for HIV infection.

## Supporting information

S1 Table Participants presented. (DOCX)
