# Supplementary material for: “I know why I am taking this pill”: Young women navigation of disclosure and support for PrEP uptake and adherence in Eastern Cape Province, South Africa
Source: PLOS Glob Public Health. 2023 Jan 20;3(1):e0000636. doi: 10.1371/journal.pgph.0000636 (PMC10021316; doi:10.1371/journal.pgph.0000636)
Supplement: S1 Table — (DOCX) [file pgph.0000636.s001.docx]

**S1 Table. Participants Presented**

| **Study Identifier Name*** | **Age** | **Adherence Level^@^** | **Site** |
| --- | --- | --- | --- |
| Avela | 22 | High | Urban |
| Lulama | 17 | High | Urban |
| Zintle | 19 | High | Rural |
| Anathi | 24 | High | Urban |
| Cwayita | 23 | High | Rural |
| Lindelwa | 20 | High | Urban |
| Fundiswa | 16 | High | Rural |
| Bongani | 23 | High | Rural |
| Ndiliswa | 22 | Low | Urban |
| Asemahle | 18 | Low | Rural |
| Babalwa | 16 | Low | Rural |
| Noziphiwo | 18 | Low | Rural |
| Akhona | 17 | Low | Rural |
| Siyanda | 18 | Low | Urban |
| Nomble | 17 | Low | Rural |
| ***** Study Identifier Names are pseudonyms and not the real names of study participants  **^@^** High adherence was defined as ≥700 fmol/DBS punch (~4-7 tablets per week); Low adherence was defined as ≤699 fmol/DBS punch (~3 or less tablets per week) | | | |
